# Pyrosequencing quantified methylation level of BRCA1 promoter as prognostic factor for survival in breast cancer patient

**DOI:** 10.18632/oncotarget.8355

**Published:** 2016-03-25

**Authors:** Feng-Feng Cai, Su Chen, Ming-Hong Wang, Xiao-Yan Lin, Lian Zhang, Jia-Xin Zhang, Lian-Xin Wang, Jun Yang, Jin-Hua Ding, Xin Pan, Zhi-Ming Shao, Ewelina Biskup

**Affiliations:** ^1^ Department of Breast Surgery, Yangpu Hospital, Tongji University School of Medicine, Shanghai, PR China; ^2^ Department of Molecular and Cellular Biology, School of Forensic Sciences, Xi'an Jiao Tong University Health Science Center, Xi'an, Shaanxi, PR China; ^3^ Department of General Practice, Yangpu Hospital, Tongji University School of Medicine, Shanghai, PR China; ^4^ Department of Breast Surgery, Fudan University Shanghai Cancer Center, Shanghai, PR China; ^5^ Department of General Surgery, Qidong Hospital, Jiangsu, PR China; ^6^ Department of Thyroid and Breast Surgery, Ningbo Medical Center Lihuili Eastern Hospital, Ningbo, Zhejiang, PR China; ^7^ Central Laboratory, Yangpu Hospital, Tongji University School of Medicine, Shanghai, PR China; ^8^ Department of Breast Surgery, Key Laboratory of Breast Cancer in Shanghai, Fudan University Shanghai Cancer Center, Department of Oncology, Shanghai Medical College, Fudan University, Shanghai, PR China; ^9^ Department of Oncology, University Hospital of Basel, Basel, Switzerland

**Keywords:** breast cancer, BRCA1, methylation, pyrosequencing, prognosis

## Abstract

BRCA1 promoter methylation is an essential epigenetic transcriptional silencing mechanism, related to breast cancer (BC) occurrence and progression. We quantified the methylation level of BRCA1 promoter and evaluated its significance as prognostic and predictive factor. BRCA1 promoter methylation level was quantified by pyrosequencing in surgical cancerous and adjacent normal specimens from 154 BC patients. A follow up of 98 months was conducted to assess the correlation between BRCA1-methylation level vs. overall survival (OS) and disease free survival (DFS). The mean methylation level in BC tissues was significantly higher (mean 32.6%; median 31.9%) than in adjacent normal samples (mean 16.2%; median 13.0%) (*P* < 0.0001). Tumor stage (R = 0.6165, *P* < 0.0001) and size (R = 0.7328, *P* < 0.0001) were significantly correlated with the methylation level. Patients with unmethylated BRCA1 had a better OS and DFS compared to the methylated group (each *P* < 0.0001). BRCA1 promoter methylation level has a statistically significance on survival in BC patients (HazR = 1.465, *P* = 0.000) and is an independent prognostic factor for OS in BC patients (HazR = 2.042, *P* = 0.000). Patients with ductal type, HER2 negative, lymph node negative stage 1+2 tumors had a better OS and DFS. Classification of grades and molecular subtypes did not show any prognostic significance. Pyrosequencing is a precise and efficient method to quantify BRCA1 promoter methylation level, with a high potential for future clinical implication, as it identifies subgroups of patients with poorer prognosis.

## INTRODUCTION

Breast cancer is the most common noncutaneous cancer and the leading cause of cancer deaths among women [1–4]. After increasing for more than 2 decades, female breast cancer incidence rates began decreasing in 2000 (due to decline in HRT), but returned to a rising tendency along with the demographical aging and manifestation of cancer risk factors in the modern lifestyle [5]. Screening techniques remain a crucial part of prevention and reduction of breast cancer mortality [1].

The breast cancer susceptibility gene 1 (BRCA1), located on chromosome 17q21, has been known since 1994 [6]. This tumor suppressor gene is a part of the DNA repair complex, which maintains genomic stability via DNA double-strand breaks repair [7]. Despite genetic heterogeneity, variations among different ethnicities and differences in penetrance, BRCA1 gene mutations have been proven related to an increased risk of hereditary breast cancer, accounting for as much as 81% [8, 9]. Thus, BRCA1 became a central tumor predisposition gene and its biology has been a subject of intense investigations [10]. Identification of BRCA1 mutations with resulting DNA repair defects, could translate into new, targeted therapeutic or even preventive approaches. So far, several hundreds of BRCA1 mutations have been found. These are mostly germline mutations, where loss of heterozygosity (LOH) is required for tumorigenesis in mutation carriers. However, a significant part of familial breast cancers are not associated with BRCA1 mutations. In those, other mechanisms cause inactivation or malfunction of BRCA1 gene [11], e.g. epigenetics. Epigenetic processes are essential for normal growth, creation and development of a phenotype [12, 13]. They also seem to be involved in tumorigenesis, causing mainly proximal, distal transcriptional and post-transcriptional non-coding changes, including DNA methylation [14–16]. DNA hypermethylation at strategic promoter regions is one of the best-characterized transcriptional-silencing phenomena, occurring at all stages of tumor development and progression [17]. Since epigenetic alterations are reversible, their modifications are an urgent target for antitumor therapy and emerged to a valuable approach in chemotherapy and chemoprevention trials of various tumor entities [18–21].

Promoter hypermethylation is the major transcriptional silencing mechanism in BRCA1, ranging from 13%–40% in sporadic breast cancer [22]. It is a tumor cell specific event in early tumorigenesis and generally absent in normal cells. It correlates with breast cancer incidence, causing BRCA1 loss of function due to a modified expression profile of the gene [23–25].

In BRCA1 mutant or silenced cells, the homologous recombination repair mechanism is defective, resulting in high sensitivity to DNA-damaging agents and poly (ADP-ribose) polymerase inhibitors (PARPi). Precisely these are the critical features, bearing therapeutic and predictive potential. Aberrant methylation of BRCA1 promoter is also associated with breast cancer occurrence. Particular biology and clinicopathology in these cancer cells result from a down-regulation of mRNA [26].

BRCA1 mutation carriers have an approximate risk of 85% to develop breast cancer. Scientifically based risk prediction for non-hereditary breast cancer is still not possible. Recent studies reported that methylation status of several genes might have a predictive value in breast cancer patients. DNA methylation markers have been found in serum and urine, and suggested to have a prognostic and predictive significance in breast cancer risk and overall prognosis prior to the diagnosis [27, 28]. Highly sensitive methods, like pyrosequencing analysis, allow an early detection and quantification of DNA methylation, which then can be used to estimate the overall survival, prognosis, therapy outcome, or response [29]. BRCA1 promoter methylation is correlated with poor overall outcomes for Caucasian and Chinese breast cancer patients [22, 30]. Triple-negative patients with BRCA1-methylated tumors however seem to be more sensitive to adjuvant chemotherapy and have a favorable survival compared to patients with BRCA1-unmethylated triple-negative tumors [31, 32, 33].

In our study, we prospectively selected 154 breast cancer patients and evaluated BRCA1 promoter methylation level in 154 paraffin-embedded breast cancer and 154 adjacent non-tumorous breast specimens. Additionally, we conducted a follow up of the patients for 98 months in order to assess the overall survival and disease free interval in BRCA1-methylated and unmethylated breast cancer patients. We used pyrosequencing analysis to precisely quantify methylation levels in matching breast tissues. To our knowledge, it is the first prospective study using quantification of BRCA1 promoter methylation in breast cancer and evaluating its relation to the overall and disease free survival. We have shown that BRCA1 promoter methylation level in breast cancer is significantly higher than that in normal tissues. We demonstrate that pyrosequencing is an efficient and effective method to measure BRCA1 methylation level in breast cancer tissues. Last but not least, we provide evidence that BRCA1 methylation is associated with a significantly poorer overall survival and disease free interval. Thus, it is a promising prognostic factor, valuable for stratifying patients, designing treatment strategies and monitoring therapy response.

## RESULTS

### Patient characteristics

Characteristics of the 154 patients enrolled in the study are summarized in Table 1. No patients died and no patients withdrew from the study after surgery. The overall characteristics distribution was comparable to general population. The follow up time was 98 months.

**Table 1:** Patients and tumor characteristics

Variables	n	%
**Age**		
<50 years	48	31.2
≥50 years	106	68.8
**Tumor size**		
<2cm	43	27.9
≥2cm	111	72.1
**Tumor stage**		
T1	28	18.1
T2	72	46.8
T3	54	35.1
**Histologic grade**		
G1	8	5.2
G2	118	76.6
G3	28	18.2
**Node status**		
Negative	89	57.8
Positive	65	42.2
**Histologic type**		
Ductal	132	85.7
Others	22	14.3
**Molecular subtypes**		
Luminal	107	69.5
Others	47	30.5
**HR status**		
Negative	46	29.9
Positive	108	70.1
**HER2 status**		
Negative	117	76.0
Positive	37	24.0
**Cancerous breast tissues BRCA1 promoter methylation**		
Negative	76	49.4
Positive	78	50.6
**Adjacent normal breast tissues BRCA1 promoter methylation**		
Negative	151	98.1
Positive	3	1.9

Abbreviations: HER2: human epidermal growth factor receptor 2; HR: hormonereceptor.

### BRCA1 promoter methylation level in cancer and adjacent tissues

**Figure 1:**
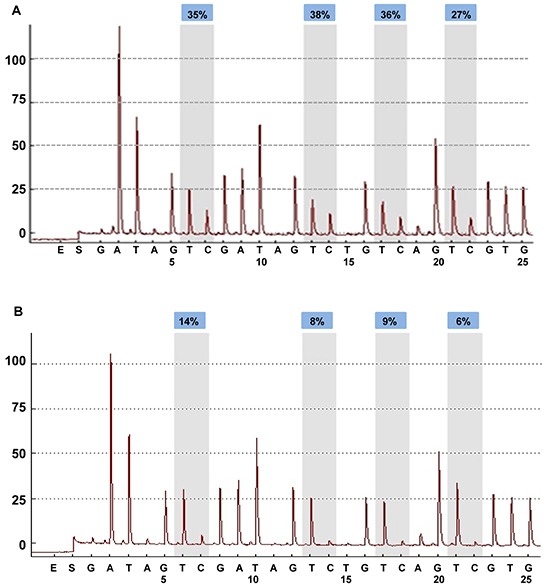
BRCA1 promoter methylation level in breast cancers quantified by pyrosequencing The percentages (blue) are the proportion of C at each CpG site after bisulfite conversion, and the methylation level of each CpG site is estimated by the proportion of C (%). An overall BRCA1 promoter methylation level is calculated as the average of the proportions of C (%) at the 4 CpG sites. Representative pyrograms: **A.** breast cancer tissues. Pyrogram of a tumor DNA showing heterogeneous levels of methylation at TC sites in the CpG island of the BRCA1 promoter. The y-axis represents the signal intensity in arbitrary units, the x-axis shows the dispensation sequence. The sequence reads GATAGTCGATAGTCTGTCAGT CGTG. **B.** adjacent normal breast tissues.

**Figure 2:**
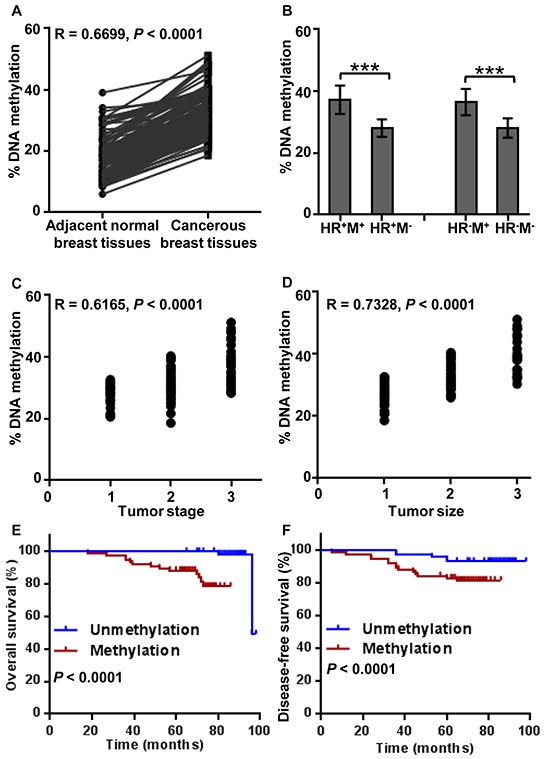
Analysis of BRCA1 promoter methylation levels' correlations Figure 2 A, B Analysis of BRCA1 promoter methylation levels in breast cancers and matched normal breast tissue. **A.** Cancer tissues showed significantly higher levels of BRCA1 promoter methylation (mean 32.6%; median 31.9%) than the adjacent normal samples (mean 16.2%; median 13%) (*P* < 0.0001). **B.** Mean level of methylation in 4 CpG sites of the promoter region of BRCA1 in hormone receptor (HR)-positive tumors were 1.33-fold (*P* < 0.0001) as compared with HR-positive unmethylated tumors, and the mean level of HR-negative methylation tumors were 1.30-fold (*P* < 0.0001) as compared with HR-negative unmethylated tumors. There was a significant correlation among BRCA1 methylation level and tumor stage **C.** and also tumor size (*P* < 0.0001) **D.** Patients with unmethylated BRCA1 promoter had better overall survival, comparing with methylated BRCA1 promoter using Kaplan–Meier method (98 months, *P* < 0.0001) **E.** Unmethylated cancer patients also had longer disease free intervals when compared to the methylated group (98 months, *P* < 0.0001) **F.**

We examined BRCA1 promoter methylation levels in 154 breast cancer tissues and their adjacent normal breast tissues. The 4 CpG sites were analyzed by methylation-sensitive pyrosequencing. Representative pyrograms are shown in Figure 1A and Figure 1B. The mean methylation level in breast cancer tissues was 34 % (Figure 1A) and 9.25 % in matching normal breast tissues (Figure 1B). As expected, cancer tissues showed significantly higher levels of BRCA1 promoter methylation (mean 32.6%; median 31.9%) than the adjacent normal samples (mean 16.2%; median 13.0%) (*P* < 0.0001, Figure 2A). BRCA1-methylation was significantly correlated with cancerous breast tissues (Rearson correlation value 0.6699 (*P* < 0.0001).

### Quantitative analysis of BRCA1 promoter methylation with pyrosequencing and relationship with clinicopathological characteristics

We analyzed the associations between the BRCA1 promoter methylation level and demographic and clinicopathological characteristics. Median age of patients with BRCA1 promoter methylation was 53 years, and 52.5 years in unmethylated patients. The mean level of BRCA1 promoter methylation in hormone receptor (HR)-positive tumors was 1.33-fold higher (*P* < 0.0001) when compared to HR-positive tumors in unmethylated patients (Figure 2B). The mean level of HR-negative methylation tumors was 1.3 times higher (*P* < 0.0001) than in HR-negative unmethylated tumors. There was no significant relationship between BRCA1 methylation level and tumor grade (R = −0.05238, *P* = 0.5188). However, there was a significant relationship among BRCA1 methylation level and tumor stage (R = 0.6165, *P* < 0.0001, Figure 2C), and tumor size (R = 0.7328, *P* < 0.0001, Figure 2D).

### BRCA1 promoter methylation vs. overall survival and disease free survival

To evaluate the clinical prognostic value of BRCA1 promoter methylation in BC patients, we categorized all patients into two groups. Patients with unmethylated BRCA1 promoter had a better overall (Kaplan–Meier method, 98 months, *P* < 0.0001, Figure 2E) and disease free survival when compared to the methylated group (98 months, *P* < 0.0001, Figure 2F).

These observations have been confirmed in univariate Cox analysis (Table 2). BRCA1 promoter methylation has a statistical significance on survival in breast cancer patients (HazR = 1.465, *P* = 0.000). Moreover, multivariate Cox analysis showed that BRCA1 promoter methylation is an independent prognostic factor in BC (HazR = 2.042, *P* = 0.000).

**Table 2:** Univariate and multivariate analysis of overall survival by the cox proportional hazards model

Clinicopathological variables	Univariate analysis			Multivariate analysis		
	HR	CI	*P* value	HR	CI	*P* value
Age	1.000	0.935-1.070	0.998	…	…	…
Tumor size	7.399	2.608-20.986	0.000	…	…	…
Tumor stage	8.803	2.542-30.483	0.001	…	…	…
Histologic grade	0.937	0.314-2.795	0.907	…	…	…
Node status	5.710	1.592-20.478	0.007	11.832	2.047-68.397	0.006^*^
Histologic type	2.642	1.229-5.678	0.013	…	…	…
Molecular subtypes	1.330	0.864-2.049	0.195	…	…	…
HR status	0.784	0.263-2.341	0.663	…	…	…
HER2 status	4.899	1.729-13.887	0.003	14.457	2.563-81.549	0.002^*^
Cancerous breast tissues BRCA1 promoter methylation	1.465	1.286-1.668	0.000	2.042	1.472-2.832	0.000^*^
Adjacent normal breast tissues BRCA1 promoter methylation	1.093	1.022-1.169	0.010	0.847	0.726-0.988	0.034^*^

… represent “date not available”;

* represent *P* < 0.05;

Abbreviations: HR, hazard ratio; CI, confidence interval.

### Overall survival and disease free survival vs. pathological type, HER2 status, lymph node status, tumor grade, stage, size and subtype

**Figure 3:**
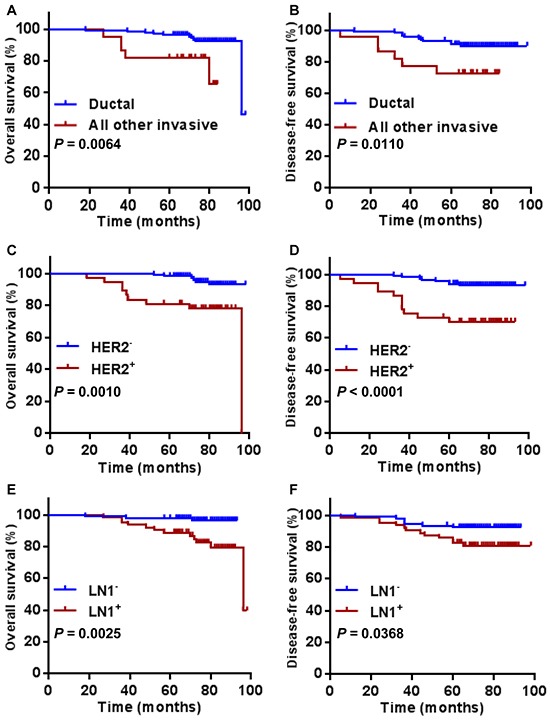
Overall survival and disease free survival according to pathological type A. and B. HER2 status C. and D. and lymph node status E. and F. Statistical significance is indicated

**Figure 4:**
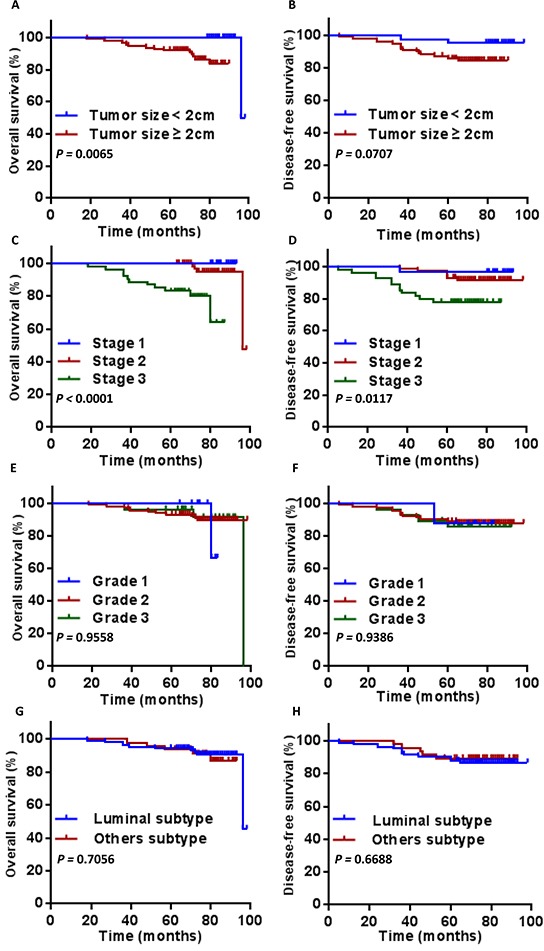
Overall survival and disease free survival according to tumor size A. and B. tumor stage C. and D. tumor grade E. and F. and subtype G. and H. Statistical significance is indicated

As expected, we observed a significantly better overall and disease free survival in ductal type (Figure 3A, *P* = 0.0064; Figure 3B, *P* = 0.0110)., HER2 negative (Figure 3C, *P* = 0.0010; Figure 3D, *P* < 0.0001), lymph node negative BC patients (Figure 3E, *P* = 0.0025; Figure 3F, *P* = 0.0368). HER2 positivity and lymph node positivity were associated with a poorer OS and DFS. Tumor size ≥ 2cm was associated with a poorer OS (Figure 4A, *P* = 0.0065), but showed no significant correlation to DFS (Figure 4B, *P* = 0.0707). Overall survival and disease free survival were significantly better in patients with stage 1 and 2 breast cancer patients, when compared to stage 3 (Figure 4C, *P* < 0.0001; Figure 4D, *P* = 0.0117). Classification of grades did not show a prognostic significance (Figure 4E, Figure 4F). Neither did the comparison of molecular subtypes of breast cancer (luminal vs. others) (Figure 4G, Figure 4H).

## DISCUSSION

DNA methylation in the BRCA1 promoter region is one of the most important epigenetic silencing mechanisms, which relates to breast cancer tumorigenesis and development. Furthermore, it is a potential indicator of chemotherapy response since a lack of a functional BRCA1 gene results in an increased tumor cells' sensitivity to molecular damage. BRCA1 promoter methylation has also been suggested as a predictor for overall and disease free survival in breast cancer patients. However, prospective, large population, long-term, follow up studies investigating the relation of BRCA1 promoter hypermethylation and patients' outcomes are scare. Statistical scientific data are lacking.

BRCA1 promoter methylation is a frequent event in sporadic breast cancers. Measurement of BRCA1 promoter methylation might thus be a new diagnostic tool [1–3]. Previous studies have shown that CpG island promoter hypermethylation of tumor suppressor genes can occur early in tumorigenesis, which has implications for early detection of cancer, particularly in people with inherited risk [34, 35]. This is an important target for oncological methodological science, since patients with BRCA1-methylated tumors have a significantly poorer disease free survival than patients with BRCA1-unmethylated tumors [36]. Especially the aggressive entity of early stage triple negative breast cancer with BRCA1 promoter methylation is associated with a poor outcome [8]. Detecting BRCA1 promoter methylation level would allow tailoring the intensity of antitumor therapy and implementing an individual treatment protocol.

To our knowledge, this is the first prospective, randomized study measuring BRCA1 promoter methylation level in breast cancer patients, comparing breast cancer and adjacent normal breast tissue, utilizing PCR pyrosequencing assay. We describe pyrosequencing as a simple, cost-effective and rapid BRCA1 promoter methylation quantification method. Our results show that pyrosequencing is efficient and can be implemented in diagnostics, therapy decision-making, vague prognostic determination and therapy monitoring. Sodium bisulfite conversion and PCR pyrosequencing assay of cancer genes methylation level allow a good precision for paraffin-embedded cancer specimens and are representative for the whole tumor [36, 37].

Most available methods for gene-specific DNA methylation analysis provide only qualitative or semi-quantitative data, which might lead to inaccurate conclusions about effects of epigenetic DNA methylation on cell cycle and metabolism. Pyrosequencing can overcome these limitations by generating highly reproducible quantification of methylation frequencies at individual consecutive CpG sites [38–41]. We demonstrated that pyrosequencing as a sequencing-by-synthesis technique is reproducible and easy, allowing a short read and a rapid measurement of BRCA1 promoter methylation level [42, 43].

Our measurements showed that BRCA1 promoter methylation levels are significantly higher in breast cancer tissues than in matched normal tissues. Interestingly, there was no significant relation between tumor grade and BRCA1 promoter methylation level. However, there was a significant relation between BRCA1 promoter methylation level and tumor stage, and size. Thus, our data indirectly confirmed that BRCA1 promoter hypermethylation was an early event of carcinogenesis in our breast cancer patients.

BRCA1-hypermethylated cases exhibited significant differences in OS or DFS when compared to the unmethylated group. This observation indicates that BRCA1 inactivation, whether by genomic or epigenetic mechanisms, is associated with a poorer patients' outcome. Strikingly, this has not been observed in ovarian cancer hypermethylation groups when compared to unmethylated BRCA1 ovarian cancer patients [33, 44, 45].

Currently, breast cancer is mostly subclassified on the basis of ER, PR, and the HER2/neu status. This classification has proven beneficial in terms of predicting prognosis and guiding treatment strategies. Yet, there is a constant need for new biological marker with predictive power, especially when one considers the heterogeneity of breast cancer. Our results are promising for the use of epigenetic information as an outcome predictor in breast cancer patients. Epigenetics has already been described as strong prognostic factor in gastrointestinal, bladder, head and neck, ovarian and several other cancer entities [19, 44, 45, 46]. Quantification of methylation levels with pyrosequencing might be useful for studying epigenetic therapy options and to determinate qualifying patients.

There are several limitations to our study. Although, to our knowledge, the patient cohort of 154 cases is the most comprehensive data composition (both genomic and clinical) assembled so far, it is still relatively small. Further studies with longer follow-ups are needed to validate our findings. We will also look into the mechanisms of BRCA1 promoter methylation specifically in breast cancer patients. Identifying new, more efficient and minimal invasive sample materials (e.g. plasma) would allow large-scale research under an easy and uniform protocol of BRCA1 promoter methylation quantification with pyrosequencing. Future work on with a higher n-number and in various ethnic groups is warranted in order to confirm that BRCA1 promoter methylation is an applicable and reliable predictive and diagnostic biomarker for breast cancer patients.

## MATERIALS AND METHODS

### Study population

We prospectively and randomly recruited 154 breast cancer patients, establishing associated clinicopathologic database and long-term clinical follow-up between January 2005 and December 2006 in the Department of Breast Surgery, Yangpu Hospital, Tongji University. All patients underwent a surgical tumor excision, in which tissue samples were collected. Preoperatively, neoadjuvant treatments of any type were not permitted.

Table 1 describes the baseline demographics of the study population. The majority of the patients presented with carcinoma of a ductal type. The median age was close to the population median age at diagnosis. The distribution of tumor grades and receptor status were representative. All patients were Chinese females and were followed until death or the end of the follow-up period.

Written consent forms were collected from all patients who were involved in this study. The ethics review board of our university approved the study design a priori.

For our experiments, we used formalin-fixed, paraffin-embedded tissues (surgically resected): 154 cancerous [classified by tumor node metastasis (TNM) stage 1: 28 cases, stage 2: 72 cases, stage 3: 54 cases] and 154 matched adjacent normal breast tissues (Table 1). Pairing normal/tumor samples allowed us to choose the value of normal samples as reference.

### Methylation analysis

We used pyrosequencing analysis both in order to study this simple and efficient method for a potential clinical use, as well as to quantitatively assess the methylation level of tissue samples and correlate them with overall survival and disease free survival of our patients.

For bisulfite conversion of the target sequences, Epitect Bisulfite Kit (QIAGEN AG, Basel, Switzerland) was used according to the manufacturer's manual. The pyrosequencing assay for DNA methylation analysis protocol has been described before [32]. Four CpG sites were analyzed by pyrosequencing using PyroMark Q96 MD (QIAGEN AG, Basel, Switzerland). In brief, double stranded PCR products were denatured with NaOH and washed before a sequencing primer was annealed. The pyrosequencing reaction started at the 3′-end of the sequencing primer. Nucleotides (A, T, C, and G) were dispensed into each sample well, one at a time. Whenever a base complementary to the base in the PCR product was added, it was incorporated into the growing DNA strand, resulting in an enzymatic cascade and production of light. The light intensity was measured at each dispensation and presented graphically in a pyrogram. The Pyromark CpG Software generated dispensation order automatically: GAT AGT CGA TAG TCT GTC AGT CGT G. Methylation data are presented as percentage of average methylation in all observed CpG sites.

### Statistical analysis

Data were analyzed by the SPSS standard version 20.0 (SPSS, Chicago, USA). The Kaplan–Meier method was used to estimate overall survival (OS), disease-free survival (DFS), and multivariate analysis was performed by the Cox proportional hazards model. OS was calculated from the date of diagnosis to the date of death or the last follow-up. DFS was calculated from the date of surgery to the date of disease relapse. The chi-square test was used to analyze the relationship between BRCA1 expression and the clinicopathological characteristics. Analysis of the differences between groups was calculated with a two-tailed Student's *t*-test. *P* values of less than 0.05 were considered statistically significant.
